# The Health Benefits of Autobiographical Writing: An Interdisciplinary Perspective

**DOI:** 10.1007/s10912-020-09631-9

**Published:** 2020-05-21

**Authors:** Jussi Valtonen

**Affiliations:** 1 Department of Psychology, New York University, 6 Washington Place, New York, NY 10003 USA; 2 Theatre Academy, University of the Arts Helsinki, P.O. Box 20, FI-00097 Helsinki, Finland; 3 Department of Psychology and Logopedics, Faculty of Medicine, University of Helsinki, P.O. Box 21, FI-00014 Helsinki, Finland

**Keywords:** Autobiographical writing, Life writing, Expressive writing, Conflict, Narratology, Psychology

## Abstract

A large body of experimental evidence in the empirical sciences shows that writing about life experiences can be beneficial for mental and physical health. While empirical data regarding the health benefits of writing interventions have been collected in numerous studies in psychology and biomedicine, this literature has remained almost entirely disconnected from scholarship in the humanities and cognitive neuropsychology. In this paper, I review the literature from psychological and biomedical writing interventions, connect these findings to views from philosophy, cognitive neuropsychology and narratology and argue that examining established regularities in how narratives are structured can shed further light on the psychological processes engaged during writing interventions. In particular, I argue that the narratological concept of conflict can be applied to resolve patterns of seemingly conflicting empirical findings in psychological studies. More generally, I propose that an interdisciplinary perspective can provide a broader theoretical basis for understanding the psychological processes underlying the health benefits of autobiographical writing and provide directions for future research in psychology and biomedicine.

*“The act of naming is the great and solemn consolation of mankind.”*

Elias Canetti, *The Agony of Flies*

## Introduction

The Nobel-winning author Imre Kertész, who had been imprisoned in Auschwitz and Buchenwald when he was young, describes writing as essential in helping him survive. Although he would carry the experience with him throughout his life, it was writing that enabled Kertész to live with the emotional and existential repercussions of the "Auschwitz disease," according to literary scholar Luísa Afonso Soares (2015). "After having written the novel I’ve almost stopped thinking about my experience in the concentration camp: it became my character’s experience. I’m free and empty," Kertész describes the writing of his novel, *Fatelessness* (cited in Soares 2015, 176).

A similar motivation was driving Shakespeare, according to philosopher Richard Kearney (2016). Shakespeare wrote *Hamlet* to mourn for his son who had died the same year and to grieve for his father who was also dying. Kearney contends that for Shakespeare, writing was a way to overcome the psychological impact of these losses to "avoid the madness of melancholy." More recently, novelist Rakesh Satyal (2018) has described how writing helped him work through experiences of loneliness, hopelessness, and romantic disappointment.

Many writers, both professional and non-professional, literary and non-literary, report that they find writing therapeutic. "In terms of cathartic effect, memoir is like therapy, the difference being that in therapy, *you* pay *them,"* according to poet and memoirist Mary Karr (2015, xxii). Rock musician Flea from the Red Hot Chili Peppers, who calls himself "completely uneducated" as a writer, says "he resorts to the act of journaling 'only when I'm really miserable,'" implying that writing helps (Pappademas 2019).

But does writing benefit well-being in ways that can be empirically demonstrated using quantitative methods? Do such benefits extend to physical health? If so, do such therapeutic benefits require specialized expertise in writing? How exactly does writing help and why?

Perhaps one of the earliest authors to recommend writing as a form of therapy was Robert Burton in his preface to *The Anatomy of Melancholy*, 'Democritus to the Reader,' first published in 1621.^1^ Writing, however, has also been studied extensively in experimental psychology and biomedicine over the past three decades. In these interventions, participants with no special training or professional experience are asked to write about their life experiences in a spontaneous fashion without revising in short sessions repeated over several consecutive days. The research on these interventions indicates that writing about life experiences can bring about impressive health benefits for people who are not professional novelists or playwrights and who do not primarily seek to provide aesthetic or artistic value to others in their writing.

How and why writing helps, however, has been difficult to explain through the lens of psychological and biomedical theories alone. While it is widely accepted in psychology that it is "the formation of a narrative" (Pennebaker and Seagal 1999, 1243) and the processing of life experiences "in a certain structured manner" (Sloan and Marx 2018, 1) that leads to the observed health benefits, psychologists rarely discuss what this structuring might be, why we tell stories about our lives, or how the generation of narratives is supported in the human brain.

In this paper, I aim to argue that the quantitative research findings from psychology and biomedicine are important in demonstrating that writing about life experiences has objectively demonstrable, quantifiable effects on well-being, but the theoretical perspective through which these findings have been interpreted in psychology is somewhat restricted. Moreover, empirical findings have called the most prominent psychological hypothesis of writing's health benefits, *the emotional disclosure account*, into question. I will argue that these difficulties can be remedied through an interdisciplinary perspective. A broader and more nuanced understanding of the writing process and its psychological value can be achieved if the psychological and biomedical findings about health outcomes are complemented by views from cognitive neuropsychology and philosophy. Moreover, the paradox of seemingly contradictory empirical findings can be resolved by applying concepts from narratology. Finally, I will suggest prospects for future research utilizing a hybrid approach combining experimental methods and narrative theory.

I will first review the evidence from psychological experiments showing that even brief writing sessions can provide psychological and somatic health benefits for lay participants. After that, I will turn to how the theoretical basis for understanding these findings can be complemented by views from cognitive neuropsychology, philosophy, and narratology.

## Does it *feel* like writing helps?

Before discussing the health benefits of writing, it is first important to note that questions of aesthetic and therapeutic value are, at least in theory, independent of each other. Arguably, writing with the aim of creating something of artistic value to others is, or can be, at least in part, a different process from when the text is written primarily for therapeutic purposes and not intended for others to read. As Robinson (2000) points out, the difference between craft and catharsis is often emphasized in literary culture. Although I believe, like Robinson (2000) and Charon (2006), that there are important aspects in which these processes often overlap psychologically, for the purposes of this paper it is mostly sufficient to assume that people can write about their life experiences without an explicit purpose of making art. The empirical evidence discussed in this paper mostly focuses on this kind of writing.

Second, it should not be taken for granted that writing necessarily confers any health benefits. Writing about life experiences could, conceivably, have no effects at all, or, especially when dealing with traumatic experiences, could even be detrimental.

In fact, dwelling on traumatic events mentally is known to be harmful for health. The tendency to ruminate on negative experiences and emotions is associated with depression (Lyubomirsky and Tkach 2004), and experimental evidence from psychology shows that spending time repeatedly thinking about negative life experiences has direct adverse effects on psychological and physical health. Lyubomirsky, Sousa and Dickerhoof (2006) conducted an experiment in which they asked twenty college students to think about their worst life experience for fifteen minutes during three consecutive days. Four weeks later, the participants reported being less satisfied with life, on average, relative to a control group of thirty-six students who had not been subjected to an intervention.

If writing has similar effects, it would thus seem better *not* to write about bad things that have happened. Is the same true, however, of writing? At least subjectively, many writers seem to disagree.

Robinson (2000) surveyed thirty-four people who considered themselves writers and who wrote regularly (albeit not all of them professionally). The vast majority, eighty-four percent, reported that they felt their writing had therapeutic value for them. Many said they had used writing to process difficult life experiences such as the loss of a close relative or other stressful periods in their lives. The same also holds for people who do not write regularly: in writing experiments conducted by psychological researchers, the majority of lay participants – with no training, experience or commitment to writing regularly – who are asked to write about their life experiences over a few repeated brief sessions report finding the experience subjectively valuable, even when instructed to write about the most painful experience in their lives (Pennebaker and Chung 2011).

Thus, many people find writing psychologically beneficial, at least subjectively, including people who both write regularly and ones who do not. This would seem to suggest, then, that something happens during the writing process that is psychologically distinct from the mere act of thinking about the same experiences. An important caveat, however, regards the subjectivity of such self-reports. A subjective feeling of psychological benefit, even among the majority, does not necessarily mean that such benefits are real or objectively demonstrable in somatic or psychological outcomes. Many interventions that appear helpful to both clinicians and patients often in reality are not (Lilienfeld et al. 2014). For this reason, psychologists have sought to investigate whether writing provides health benefits that can be demonstrated relative to an experimental control group.

## The expressive writing paradigm

To that end, Lyubomirsky et al. (2006) – in the same experiment discussed earlier – assigned yet a different group of twenty students randomly to a third condition in which they were asked to *write,* as opposed to *think,* about their worst life experience for the same duration on the same number of sessions as the thinkers.

Their findings show that writing about negative life experiences has strikingly different psychological effects from merely thinking about them: four weeks later, the writing group reported higher life satisfaction, better overall and mental health, better social functioning and fewer physical health symptoms relative to the thinking group. That is, the participants who had written about their *worst* experience felt *better* about their lives four weeks later in practically every possible way than the ones who had spent the same amount of time thinking about their experiences. Importantly, as the participants were randomly assigned to the thinking, writing and control groups, it would be difficult to attribute the differences in mean outcomes among the groups to chance alone.^2^

The experiment by Lyubomirsky et al. (2006) is one among more than two hundred published studies in psychology and biomedicine that have investigated the psychological and somatic effects of (non-literary) life writing by non-professional participants. In the experimental paradigm originally developed by Pennebaker (1997) and known in psychology as *expressive writing*, participants are randomly assigned to two groups, and both complete a writing exercise over short sessions (typically fifteen to twenty minutes) across three to five days. Participants in the expressive-writing group are instructed to write about their emotions concerning an upsetting or traumatic life event. The control group, in contrast, is given a topic considered "emotionally neutral" or "superficial" such as how participants plan to use their time during the following week but for an equal number and duration of sessions.

Researchers using this paradigm have consistently found, through quantitative measures and focus on group-level effects, that writing about emotionally upsetting life experiences can have a wide range of beneficial effects on health outcomes. Relative to group averages in the control condition, the expressive-writing participants have been found to experience a multitude of health benefits, such as improved subjective well-being, reduced use of health care services, reduced absences from work and improvements in immune function (reviews Arigo and Smyth 2016; Pennebaker 1997; Pennebaker and Seagal 1999; Pennebaker and Chung 2011; Sloan and Marx 2004). Meta-analyses indicate that expressive writing is consistently associated with positive outcomes and clinically meaningful effect sizes across studies (Frattaroli 2006; Frisina, Borod and Lepore 2004; Smyth 1998; Travagin, Margola and Revenson 2015). This means that the differences (in group means) between the writing and control group participants are not only statistically significant but sufficient to be likely to be meaningful for the participants' real lives.

In addition to outcomes related to psychological well-being (Lyubomirsky, Sousa and Dickerhoof 2006; Murray and Segal 1994), expressive writing interventions have been found to benefit physical health. For example, Smyth, Stone, Hurewitz and Kaell (1999) randomly assigned one hundred twelve patients with asthma and rheumatoid arthritis into two groups, one instructed to write about the most stressful experience of their lives and a control group about their plans for the day. Their clinical condition was evaluated with spirometry and in a rheumatological evaluation in a blinded setting (i.e., the clinical examiners did not know which one of the two writing assignments an individual patient had completed).

While there was no change in the control group's disease state, the asthma patients who had written about their stressful experiences showed an improvement in lung function relative to a pre-intervention assessment, as measured in forced expiratory volume four months after the writing intervention. Similarly, there was no change in the disease state in the group of rheumatoid arthritis patients who had written about their plans for the day; in contrast, the rheumatoid arthritis patients who wrote about a stressful life experience showed a clinically relevant change in their health status four months after the intervention. Of all patients in both disease groups, 47% of those in the expressive writing condition showed a clinically relevant improvement in their disease state, as opposed to only 24% of those who completed the control assignment. That is, while the clinical status of some patients also improved in the control group, this was true of a significantly fewer number of patients than in the writing group.

Other studies have found expressive writing interventions, for example, to reduce viral load and increase lymphocyte counts in HIV patients (Petrie et al. 2004); to lower Epstein-Barr virus antibody titers (Esterling et al. 1994); to increase the level of antibodies against a hepatitis B vaccine (Petrie et al. 1995); to improve liver function (Francis and Pennebaker 1992); to improve the state of the disease in irritable bowel syndrome (Halpert, Rybin and Doros 2010); to relieve pain in fibromyalgia patients (Broderick, Junghaenel and Schwartz 2005); to reduce health care utilization among healthy populations (Harris 2006); and to provide a host of other health benefits in various medical conditions such as cancer, sleep disorders and chronic pain (Baikie and Wilhelm 2005).

Thus, even brief writing sessions can provide surprisingly substantial changes not only in the writers' psychological but also their physical well-being, according to the empirical biomedical evidence. Because of the impressive somatic health effects, an editorial in the *Journal of the American Medical Association* (JAMA) contends that were there similar outcome evidence about a new drug, "it likely would be in widespread use within a short time" (Spiegel 1999, 1329).

While these findings are arguably impressive and important, they of course do not show that writing benefits everyone or explain why writing helps. What the psychological and biomedical group studies show is that putting life experiences into words on paper is beneficial for a sufficient number of lay participants for group-level effects to emerge statistically. The quantitative group averages in these data do not tell us who benefits and who doesn't, or why some people benefit more than others although psychological researchers have made efforts to disentangle some of these questions (see Pennebaker and Chung 2011). These quantitative data similarly do not tell us to which extent the participants' writing was shapely, refined, or coherent, to which extent it may have had aesthetic value, or whether attempts to create art from the experiences might affect writers differently. They only provide evidence that *something* happens during the writing that is psychologically beneficial to a large number of participants relative to the comparison conditions.

Why, then, does writing help? Despite the mounting empirical evidence, the psychological processes underlying these health benefits have remained somewhat unclear.

## What is it about writing that helps?

Psychological and biomedical researchers have proposed several hypotheses to explain why writing helps. In psychology, these have included those based on Freudian, cognitive, learning theory, self-regulation, and social-connections approaches, among others (see Frattaroli 2006; Pennebaker and Seagal 1999; Sloan and Marx 2004; Pennebaker and Chung 2011).

Perhaps the most prominent category of psychological explanation is based on the notion that the disruptive consequences of a stressful life experience come from two sources: first, the negative experience itself can be detrimental for well-being, and second, any negative effects will be further exacerbated if the experience is kept a secret. What is assumed to help in the expressive writing condition, then, relative to the use-of-time writing condition, is the "emotional disclosure" (or verbal "confrontation") of the traumatic event. Practically all proposed hypotheses in psychology assume, in the words of Sloan and Marx (2018, 1), that "confronting a previously avoided stressful or traumatic life event in a certain structured manner" is what helps.

Biomedical authors, in contrast, have focused on proposed biomedical pathways mediating psychological and somatic effects. Spiegel (1999) suggests that writing may dampen the effects of stress on the body through the operation of the hypothalamic-pituitary-adrenal axis (HPA) – a suggestion not incompatible with perhaps any of the psychological hypotheses. In yet a third perspective, social neuroscientists have proposed that writing may ameliorate affective distress through activating specialized neural networks in the right ventrolateral prefrontal cortex in the brain (RVLPFC) (Lieberman 2011).

While these hypotheses are not without merit, they also appear limited. It is widely agreed that no single theoretical perspective can explain the strikingly wide range of effects (King 2001; Pennebaker 2004; Sloan and Marx 2004). Different benefits have been reported for bereaved individuals, cancer patients, prison inmates, individuals taking exams, people suffering from migraines, and female caregivers (Frattaroli 2006; Pennebaker 1997; Sloan and Marx 2004; Smyth 1998). In one study, unemployed engineers found employment faster after a writing exercise (Spera, Buhrfeind and Pennebaker 1994); in another, romantic couples were more likely to stay together (Slatcher and Pennebaker 2006); in yet a third, students preparing for graduate school exams had higher test scores and lower rates of depressive symptoms than those in the control group (Frattaroli, Thomas and Lyubomirsky 2011).

For these reasons and others, psychologists have lamented on the lack of theoretical insight into the writing process and its effects. According to Sloan and Marx, "The entire genre of expressive writing research has suffered to some extent from its lack of systematic focus and weak theoretical foundation" (2018, 4). In the words of psychologist Laura King: "Two strong conclusions can be made with regard to the benefits of writing. First, expressive writing has health benefits. Second, no one really knows why" (2001, 119).

My proposal is that these problems stem in part from an unnecessarily narrow empirical perspective in psychology. The experimental approach, centered on collecting quantitative data sets from large groups of participants, is well suited for answering particular kinds of questions. Through systematically manipulating experimental settings, this research has furthered our understanding of the significance of individual variables such as the timing of the writing sessions: one-week intervals between writing sessions are associated with larger effect sizes, on average, than daily writing sessions (Pennebaker and Chung 2011; Smyth 1998).

The psychological and biomedical literature on writing interventions has, however, remained almost completely disconnected from relevant bodies of scholarship in cognitive neuropsychology, on the one hand, and disciplines in the humanities on the other that work to understand how narratives are structured. An interdisciplinary perspective can therefore alleviate these shortcomings.

## Narrative and cognition: stories are how humans make sense of experiences

Why might writing be helpful for well-being? Although this research is rarely cited in the context of expressive writing in psychology, established views both in the humanities and in cognitive neuropsychology propose that constructing stories is a fundamental way in which we understand our lives.

Literary critics and philosophers have argued for a long time that narratives are psychologically essential for how humans make sense of their experiences. According to bioethicist Howard Brody, "The primary human mechanism for attaching meaning to particular experiences is to tell stories about them" (2002, 13). The literary critic Frederick Jameson calls narrative the "central function or instance of the human mind" (cited in Abbott 2008, 1). Sociologist Arthur Frank (1995) contends that stories "are the self's medium of being"; it is a "rage for order," in the famous words of poet Wallace Stevens. According to literary scholar H. Porter Abbott, "The gift of narrative is so pervasive and universal that there are those who strongly suggest that narrative is a 'deep structure,' a human capacity genetically hard-wired into our minds in the same way as our capacity for grammar (according to some linguists) is something we are born with" (2008, 3).

These views are highly compatible with prominent discoveries in contemporary neuropsychology, showing that the human brain houses a cognitive subsystem specialized for making sense of unexpected events by generating narratives. Experiments with neurological patients whose interhemispheric connections have been disconnected suggest that the left hemisphere of the brain supports a system of specialized cognitive processes that enable the generation of hypothetical narratives for events and experiences that demand explanation (Gazzaniga 2000). Interestingly, this line of work has not, to my knowledge, been discussed among psychological or biomedical researchers seeking to understand the why writing interventions help.

Cognitive neuroscientist Michael Gazzaniga and colleagues have named this left-hemisphere supported process "the interpreter". The left-brain interpreter is, according to Gazzaniga, "[a] device with rules for figuring out how one thing relates to another. … Its job is to interpret our responses—cognitive or emotional—to what we encounter in our environment. The interpreter sustains a running narrative of our actions, emotions, thoughts, and dreams" (2000, 1320). Gazzaniga argues that the left-hemisphere-enabled process of crafting narratives is what forms the basis for our subjective unified feeling of being and, thus, an essential part of the human condition. According to him, "The interpreter is the glue that keeps our story unified and creates our sense of being a coherent, rational agent." To our collection "of individual instincts it brings theories about our life. These narratives of our past behaviour seep into our awareness and give us an autobiography."

Gazzaniga's neuroscientific view of narrative as a unifying glue that underlies our sense of being is consistent with the concept of *narrative identity* proposed by the philosopher Paul Ricoeur. According to Ricoeur, narrative identity is "the kind of identity that human beings acquire through the mediation of the narrative function" (1991a, 188).

The notion of a cognitive module in the human brain supporting a function centered primarily on making sense of unexpected events is also congruous with scholarship on established regularities in how narratives are typically structured. In Gazzaniga's experiments, the split-brain patients were subjected to conditions in which something violated their assumptions or their experience; their perceptual input typically conflicted with their expectations or other aspects of their experience. Narratologists argue that this is what a story worth telling typically requires – an event that violates the expected script of events and disrupts an initial state of equilibrium. What narrative theorists refer to as *the point of attack* is what initiates the action line of a story (Herman, Jahn and Ryan 2005; also referred to as the "precipitating event," Bruner 1991). In Gazzaniga’s studies, the experimenters deliberately violated the expected script of events for the patients, which prompted them (using only the left hemisphere of their brain) to generate verbal narratives to make sense of the situation.

Thus, both experimental neuropsychologists and philosophers agree that narrative is an essentially human medium of existence, one by which we seek to understand unexpected, untoward and problematic events.

## Problems with psychological accounts of writing's health benefits

While integrating views from philosophy and cognitive neuropsychology helps to broaden the theoretical basis for understanding why writing might benefit well-being, there are also more specific problems with the psychological *emotional disclosure account* of the health benefits of writing. I will argue that these problems also arise from an overly limited empirical viewpoint in psychology that fails to acknowledge what narratives are and how they typically work.

The first problem is theoretical. As mentioned earlier, the most prominent psychological accounts assume that it is the "emotional disclosure" of traumatic experiences that is helpful in writing. And, indeed, the account seems consistent with Pennebaker's (1997) original expressive writing experiments in which the participants were asked to write about a negative, traumatic or stressful life event and to focus on the related emotions.

The precise difference between the expressive-writing and control conditions, however, is important: The health benefits that emerge are always demonstrated relative to the control writing assignment – such as how the participants organize their use of time for the week. The evidence about the benefits of writing are demonstrated as a difference in group averages as compared to writing about this other topic. In the psychological literature, the control topic is often described as "superficial" or "emotionally neutral." The emotional disclosure account proposes that the traumatic-life-experiences topic leads participants to a process of written "emotional disclosure," whereas the "superficial" topic doesn't. But what is it, exactly, that makes a writing topic "superficial" and why? The concepts used to define the control condition are surprisingly vague in the psychological literature, despite their centrality for the interpretation of the findings.

One reason the conceptual haziness is problematic is that it makes the emotional-disclosure explanation somewhat circular. The writing instruction already uses emotional vocabulary ("traumatic" or "negative" life experiences), and writing, by definition, means disclosure. (The texts in the expressive writing paradigm are typically not written for anyone else to read.) The emotional disclosure account seems to contend, then, that the disclosure of emotionally difficult experiences helps because it involves the disclosure of emotionally difficult experiences. To avoid this circularity, it is essential to define the conceptual difference between the two writing tasks compared.

In addition to this theoretical problem, there is also a specific empirical problem with the emotional disclosure account. That is, the health benefits of writing are not, according to research findings, limited to assignments in which participants are asked to write about their emotions related to negative life events. While the participants were all asked to do so in Pennebaker's original experiments, psychologist Laura King (2001) later showed that a (seemingly) completely different writing assignment surprisingly also led to similar group-averaged health benefits as in the trauma-disclosure experiments, one that does not require emotional disclosure of any troubling experiences at all.

In King's experiment (2001), one group of participants again wrote about a traumatic life event, whereas a control group wrote about how they used their time. Critically, however, King also randomized an additional third group of participants to write about their life goals. That is, instead of a stressful or traumatic experience, the participants were asked to imagine almost the opposite: how everything in their life in the future has gone as *well* as possible and to write about that. The life-goals assignment does not seem related to traumatic events or their emotional disclosure at all. Despite the difference in assignments, however, both the participants assigned to the life-goals group and to the trauma-writing group made significantly fewer visits to the health center during the next five months than those in the control group (whose visits tended to slightly increase). In other words, writing about life goals was equally beneficial to the writers' health as the disclosure of emotional trauma.

The similar health benefits both groups received, despite the different writing tasks, have puzzled psychologists because the findings are at odds with the emotional disclosure account. Analyses further showed that the participants in the trauma and life-goals groups not only followed different instructions but also wrote differently: groups of independent (lay) raters judged the texts of the trauma-group participants to be more emotional, more negative, and to attribute responsibility to others more than texts written about life goals. Thus, both the instructions and the texts were different in content, yet the health benefits were similar.

Why were both assignments advantageous for the writers' health? And why was writing about the use of time not? Why is writing about the use of one's time a "superficial" topic but writing about life goals (presumably) not? How can we avoid the circularity of defining any task that fails to help as "superficial" and the ones that do as not?

## A narratological resolution to a psychological paradox

To seek for answers, it is informative to examine a text written by a research participant in the life-goals group in King's experiment:

I have learned to love as fully and selflessly as possible. I have learned to be humble without losing and confidence, esteem, or being fake. I have touched the life of at least one other soul in a significant way and helped them learn to love more greatly. If I have achieved this then I know that I have also achieved happiness, peace, and worldly success. By this last I refer to career accomplishment, and enough material wealth to keep me satisfied. . . . I have learned to relinquish all fear. (2001, 802)

Narrative theorists have identified a number of *narrative universals,* elements or features that tend to recur in stories (Hogan 2005), which can complement the lack of theoretical perspective in the psychological research literature. Although psychological researchers do not discuss their experiments in these terms, King's assignment directs participants towards a well-identified narrative universal related to character, *a hero striving for a goal.*

Strictly speaking, King's participant's text is a non-narrative text, a description of a state, rather than a narrative. According to Prince's definition, a minimal story consists of three events that are in temporal succession, causally related and bring about some form of closure, such as an inversion of the original state: "He was rich, then he lost lots of money, then, as a result, he was poor" (1973). King's participant's text does not describe such a succession of events. Importantly, however, the text does not exist in isolation, but in the context of the instructions:

Think about your life in the future. Imagine that everything has gone as well as it possibly could. You have worked hard and succeeded at accomplishing all of your life goals. Think of this as the realization of all of your life dreams. Now, write about what you imagined.

Interpreted within the context of the instructions, the participant's text has not only a protagonist but also a beginning and an end – that is, events in temporal succession. The temporal succession and the change in the original state – the requirements of a narrative – are implied by the combination of a protagonist striving for life goals and the goal states described in the text that the protagonist reaches. The original state in the story is the protagonist's present reality.

While it is not possible to disambiguate all the text's indeterminacies because most of the story's constituent events are left out, the narrator does describe the end states of multiple story-lines in the protagonist's life: the protagonist has learned not only to love but to love *in a certain way*, learned to be humble *in a specific way*, has touched the life of another soul in a significant way, and so on. We do not know whether or not the multiple story-lines intersect, nor do we know whether one of them is the main story-line to which the others are subsidiary. For each of the story-lines, only the resolution is provided.

A hero can only strive for a life goal, however, if the goal is meaningfully different from the state from which they begin. If the resolution of the first story-line is, "I have learned to love as fully and selflessly as possible," the initial state is presumably something non-trivially unlike this state, possibly its inversion: the protagonist has been unable in the beginning, in some important way, to "love fully and selflessly." Otherwise there would be no striving or goals to be reached (and hence, no goals to write about). The narrator would similarly not consider "learning to be humble without… being fake," an important life goal if this state had already been reached or if this were trivially easy to achieve. According to this interpretation, then, the protagonist is first unable to love fully and selflessly and then learns to do so.

What could cause such a change in a person’s life? In a paradigmatic story arc, the driving force propelling a story forward is *conflict*, "the thwarting of intended actions by unplanned events, which may or may not be the effect of other characters' intended actions" (Herman 2005b). As Aristotle pointed out, readers and writers tend to expect narratives to begin from an original state of equilibrium and advance through a phase of conflict to an ending in which a new equilibrium is achieved (Herman 2005a, 2005b; Prince 1973; Rimmon-Kenan 2002). While the gappiness of the text allows for numerous possibilities, it is clear that the conflict in this story-line will, in any interpretation, revolve around the inability "to love fully and selflessly," which apparently has proven more challenging than the protagonist initially assumed.

The possibilities for how this conflict occurs and what the constituent events may be are, of course, innumerable, but choosing this as a central life goal clearly suggests that the protagonist is conscious of at least some shortcomings in this area – something has violated the protagonist’s initial state of equilibrium and led to conflict in one way or another. Perhaps the protagonist has countered a relationship that forced him or her to recognize that loving fully and selflessly can be complicated and demanding; perhaps the protagonist has struggled to reconcile his or her own desires with societal expectations; perhaps he or she has experienced failed attempts at balancing another person's values, needs and desires with one’s own. Whatever the details may be, the struggle for a new equilibrium is based on the recognition that loving fully is a skill the protagonist does not currently possess but needs to acquire.

Thus, the struggle for "learning to love as fully and selflessly as possible" is a critical theme in the protagonist's life, *in any interpretation* of this text. The same is naturally true of all the other story-lines in the text: each of them individuates a goal state that has not yet been reached and involves a meaningful struggle ("you have worked hard") related to a theme that is important. The protagonist in King's participant's text would not be striving for the realization of these life dreams unless they were both important and difficult – that is, if they did not involve conflict and have exceptional personal value. It is pointless to dream of life goals that are meaningless, trivially easy to achieve or have already been accomplished.

Thus, considered from the viewpoint of narrative, the life-goals text is not only a description of ideal states but also contains implications of story-lines that all involve conflict centered on important life challenges. Psychologically, the life-goals writing assignment likely guides participants to explore and mentally simulate ideal resolutions to important conflicts in their lives – to put into written words their imagined ideal states of denouement for conflicts in life they consider most important.

### A paradox resolved: narrative and conflict

This simple application of a narratological perspective to a participant's text from King's experiment provides important implications for the interpretation of the results. The main insight is this: although the participants were neither instructed to explore sources of psychological conflict nor asked to explain which conflicts they considered important in their lives, this may very well be what they did.

Experimental psychological studies that focus on intervention outcomes reveal little about what goes on in the minds of individual writers. Stories, however, are highly structured entities governed by established regularities (e.g., Rimmon-Kenan, 2002), and there are shared elements among humans in the ways we (typically) relate to narratives. These shared elements guide readers in making sense of texts, regardless of our other views and beliefs about the world (Abbott 2008). The above examination of one participant's text can therefore shed light into what may have occurred during its writing psychologically.

While this analysis may not sound groundbreakingly insightful or innovative from a narratological point view, it may help to explain why the life-goals writing assignment led to similar health outcomes as writing about traumatic events in psychological experiments. Conflict is a narrative universal that participants (including those without a literary background) will have encountered in countless stories regardless of genre, both as readers and as writers, and it is therefore not surprising that it can emerge also in narratives constructed during instructed interventions, including ones about life goals when the assignment allows.

Literary authors often describe writing as a process of discovery. The novelist Margaret Atwood describes writing as a desire to enter a kind of darkness, "and, with luck, to illuminate it, and to bring something back out into the light" (2003, xxii). Gillie Bolton uses a similar simile, comparing writing to "dropping a bucket into the well of oneself, pulling it up dripping to see what is there" (1999, 120); or, Seamus Heaney in his poem, "Personal Helicon": "I rhyme / To see myself, to set the darkness echoing" (1966, 57).

A possible hypothesis, then, emerging in combination from these two perspectives, is that writing is a method that can allow a writer to discover and explore important sources of conflict in their lives and to reflect on their meaning. Perhaps the participants in King's experiments were not fully aware of the central sources of conflict in their lives or their significance before beginning writing. From this viewpoint, both Pennebaker's (1997) and King's writing assignments may function in this profoundly similar way: both involve mentally identifying sources of conflict in the writer's life that are subjectively important and exploring their ramifications and possible resolutions. From this perspective, it is perhaps not surprising that the associated health benefits are similar, as different as the traumatic-event and the life-goals writing assignments superficially appear.

An important additional point psychologically is that the text a participant writes during such an intervention is not nearly as ambiguous to the *author* as to the reader. Although an outside reader cannot disambiguate the innumerous possibilities, the author will mentally fill in the gaps and process the implied conflicts in ways that are far richer. It is therefore reasonable to assume that during the writing process, the writer will mentally process the challenges related to what they value in life in complex and intricate ways and continue to do so in the aftermath of the writing.

The suggestion that conflict is central for the health effects of expressive writing is further corroborated by other studies. Positive health outcomes have been reported also when unemployed engineers wrote about the experience of losing their jobs –another way of guiding participants to craft written narratives centered on conflict central in the writers' lives. In yet another writing assignment, similar health benefits were found when participants were asked to write about an *imagined* trauma (Pennebaker and Seagal 1999). Again, the writers were directed toward sources of conflict that they have either experienced or can imagine could threaten things important in their lives. Although some assignments ask that the participants remember the past (past trauma, past lost job) and others that they imagine the future (life-goals, imagined trauma), they all require the writer to identify and explore, from the distance of a written narrative, conflicts that feel subjectively both important and challenging in the present.

This may be the essential reason that the using-your-time assignment is different from the negative-experiences, imagined-trauma and life-goals assignments: writing about how to use your time this week is not as likely to lead to an examination of subjectively meaningful sources of important conflict in life. Psychologists are thus correct in describing it as "superficial" topic, but grounding the comparison in the narratological concept of conflict and established regularities in how narratives function provides a theoretical basis for understanding why that is.

## Why does writing help? Conflict and psychological resolution

The previous analysis suggests that there is something psychologically particularly relevant about conflict in narrative. Why is conflict so central to what we spontaneously write about? Why is it such an essential component in stories we generally prefer to read? Without conflict, events either predictably follow an expected path or feel unimportant (Bruner 1991; Herman 2005b). Some screenwriters go as far as to insist that conflict is required in every single scene for a story to remain interesting to the audience (Ackerman 2003).

Novelist Nellie Hermann notes that people often end up writing about conflict regardless of assignment type. Even when she uses very general prompts with clinicians, it is almost as if they are waiting for an opportunity to write about the important conflicts in their lives:

…this same work is valuable for all of us, even with all of our daily disruptions, the traumas that are not so great that they disrupt the flow of our lives. … And it is not uncommon for traumatic events to come to the surface very quickly in response to a writing prompt—I have never done a workshop with medical professionals where at least one vivid traumatic event has not poured onto the page in the 3-minute window they have to write. I am always amazed at the memories that are called back in response to a writing prompt, decades-old memories leaping to mind and to the page in less than a minute. (2016, 222)

Conflict has consistently been in the heart of all human narratives throughout history. Abbott contends that it probably performs important social and cultural functions:

One very plausible possibility is that the representation of conflict in narrative provides a way for a culture to talk to itself about, and possibly resolve, conflicts that threaten to fracture it (or at least make living difficult). In this view of narrative, its conflicts are not solely about particular characters (or entities). Also in conflict, and riding on top of the conflict of narrative entities, are conflicts regarding values, ideas, feelings, and ways of seeing the world. There is, of course, no culture without many such conflicts. Narrative may, then, play an important social role as a vehicle for making the case for one side or another in a conflict, or for negotiating the claims of the opposing sides, or simply for providing a way for people to live with a conflict that is irreconcilable (as, for example, the conflict between the desire to live and the knowledge that we have to die). (2008, 55)

These social and cultural functions are fundamentally connected to individual psychological reality. Narrative structures support human thinking by performing several important cognitive functions, such as assisting problem solving (Herman 2013). Telling stories centered around conflict would likely not play such important societal functions if they were not also a meaningful psychological tool for human thinking at the individual level.

Because of the way narratives are centered around conflict, Abbott calls narratives "a form of passionate thought" (2008, 195): readers make sense of conflict in narratives by relating them to ones they have experienced, observed or imagined in their own lives. This makes the reader concerned for, and engaged in, how the psychological, social, and cultural aspects of conflict are resolved that are at play in a narrative, and makes our emotional responses closely tied to our interpretations of the narrative. According to Abbott:

[I]nsofar as we share in our own lives the larger conflicts of which these narrative conflicts are particular examples, we are moved by the narrative, drawn into it, and become alert to how these conflicts play out. And this, I am arguing, is an important form of thinking, whether or not the negotiation of conflicts is seen to be successful. Moreover, in contrast to more abstract modes of thought, this is *passionate thinking*. That is, in narrative our thinking is intimately tied to the emotions aroused during our narrative journey. (2008, 198-199)

Perhaps, then, constructing a written narrative about stressful life experiences – or about how life goals might be accomplished in the future – provides a mode of thought, of passionate thinking, that is psychologically powerful in the face of important life conflicts, in a way that the mere act of thinking is not.

## Why does writing about life experiences help? An interdisciplinary perspective

We can now return to the essential question posed in the beginning: why is writing about life experiences and goals helpful for well-being whereas merely thinking about them is not? Although both literary scholars (Ricoeur 1991a; Abbott 2008) and cognitive neuropsychologists (Gazzaniga 2000) agree that constructing narratives is an essential mental process through which human beings make sense of their experiences, constructing written or spoken narratives seems to provide a powerful tool for processing conflict, whereas constructing mental narratives does not. Why might this be? Several possibilities offer themselves in light the prior analyses, several of which can be simultaneously true.

One suggestion is that the construction of a narrative that is written down or spoken out loud provides *distance* to the depicted experiences in a way that the construction of a mental narrative for some reason does not. According to Brody, we not only have a need to tell stories about our experiences, but the act of telling these stories also provides us with a source of self-knowledge because of the combination of *distance* and *intimacy* that personal narratives offer. "Distance exists because the narrator is separated from the narrated events in time and thus can assume a reflective, observant posture toward those events in a way that was impossible when the events were in progress. Intimacy exists, especially in autobiographical narrative, because the narrator *is* the individual mentioned in the narrative, is responsible for the events disclosed, and thus has a personal stake in how others react to the telling of the story" (2002, 25).

Pioneer of narrative medicine, Rita Charon, uses the term *autobiographical gap* to conceptualize this distance created when writing. "Any time a person writes about himself or herself, a space is created between the person doing the writing and the person doing the living, even though, of course, these two people are identical. Called the “autobiographical gap,” this space between the narrator-who-writes and the protagonist-who-acts confers the very powerful distance of reflection, without which no one can consider his or her own actions, thoughts, or life. Within this reflective space, one beholds and considers the self in a heightened way, revealing fresh knowledge about its coherent existence" (2006, 70).

Sociologist Arthur Frank (1995) notes that when abrupt, traumatic changes happen in life, people are in particular need of telling stories about their life. Gazzaniga's (2000) experiments suggest that the left-brain interpreter is typically activated when we are confronted with events that are unexpected, problematic or difficult to understand. Conceivably, the more important and the more complex the conflict one seeks to make sense of, the more helpful the construction of a narrative may be. In writing about one's life, one does not "set out to report on the self but rather to eavesdrop on oneself, writing about it so as to learn what it might have become," according to Charon (2006, 72).

A further degree of complexity comes from the observation that life experiences can be, in turn, shaped and sometimes also distorted by the act of arranging them into a written narrative. An interesting question – that psychological and biomedical researchers have so far not attempted to address – concerns whether the health benefits of writing may be related to the degree to which the writer is or isn't telling the truth about their experiences. Some writers, however, like poet and memoirist Mary Karr (2015), argue vehemently that there is particular psychological (and artistic) value in confronting one's false beliefs and forms of self-deception through the writing process.

The act of constructing a narrative can also be more consequential than it may seem: As Charon points out, "Telling our story does not merely document who we are; it helps to make us who we are" (2006, 69). It is a constructive act which, according to the poet Dannie Abse (1998), profoundly affects, or perhaps even redirects, the trajectory of the writer's life itself. Following Aristotle, Paul Ricoeur (1991b, 23) talks about *phronetic* or narrative understanding, a form of knowledge stories can provide that is closer to "the practical wisdom of moral judgment" than to scientific knowledge or theoretical reasoning. Arthur Frank (2004) similarly argues that stories can help us develop *phronesis,* wisdom about life that can only come through a combination of experience and reflection. While writing about life experiences may reduce the number of visits to the doctor's office (Pennebaker and Chung 2011), the process can be even more valuable in providing a deeper view of the meaning of one's experiences, one's values and possible future selves.

## Future directions

James W. Pennebaker, the pioneer of the expressive writing paradigm in psychology, contends that writing affects people on multiple levels, including (at least) the cognitive, emotional, social, and the biological, and over both the short and the long term; writing "touches all parts of [writers'] lives" (2004, 140).

In this paper, I have aimed to argue that the positive health outcomes of expressive writing interventions in psychology and biomedicine can be best understood from an interdisciplinary perspective, broadened by views from philosophy, cognitive neuropsychology and narrative theory and grounded in an understanding of the established regularities in how narratives typically function.

The concept of conflict and the central role it plays in narrative is merely an example of the kinds of conceptual tools experimental psychologists and biomedical researchers could have at their disposal utilizing perspectives from the humanities. The application of other narratological concepts and methods to psychological writing interventions could potentially also shed similar light into other ways in which writers make meaning of their experiences during the writing process.

For example, psychological experiments suggest that the participants who are able to construct coherent stories from their experiences during the writing process are more likely to benefit from it than those who are not. Pennebaker and Chung (2011) note that participants who begin a psychological writing intervention with a coherent story explaining their past experience "generally do not benefit from writing," unlike participants who generate such a story during the process. Coherence in writing, however, has proven difficult for psychologists to assess and investigate:

"Unfortunately, we are not yet at the point of being able to precisely define what is meant by coherent, understandable, or meaningful when it comes to writing about emotional upheavals," Pennebaker and Chung confess (23). "One person’s meaning may be another’s rumination. Many times in our own research we have been struck by how a person appears to be writing in a way that avoids dealing with what we see as a central issue. Nevertheless, the person’s health improves and he or she exclaims how beneficial the study was. Meaning, then, may ultimately be in the eyes of the writer."

How meaning emerges from texts through interpretation, however, is an area extensively studied by narratologists and other narrative theorists. While meaning is, in some ways, in the eyes of the writer, in other important ways it is not (e.g., Rimmon-Kenan 2002; Bruner 1991). Scholarship from the humanities could importantly complement the experimental methods used by psychologists to provide insights into the interpretation of the participants' texts – material research psychologists rarely examine (aside from isolated linguistic components detached from their context) – and into the underlying psychological processes.

For example, several authors have pointed out that metaphor is an important tool by which humans organize their thought processes (e.g., Hesse 2000; Ricoeur 1977, 1991b). The roles that narrative phenomena such as metaphor play in writing have not, to my knowledge, been examined in psychology – perhaps because they depend on context and are not easily quantified mechanically across large groups of participants, as opposed to, for example, the frequencies of first-person pronouns in texts. Such phenomena may be difficult or even impossible to isolate experimentally, but it would be possible to take an interdisciplinary perspective and look for generalizable patterns across participants in the ways such literary devices and others are (or are not) used in autobiographical writing.

A combined approach informed by both narratological and experimental methods would enable interdisciplinary scholars to probe questions about psychological processes at a deeper level and ask questions such as (but not limited to): Are the ways in which writers represent conflict in their writing related to identifiable patterns of health outcomes? Is the presence of a particular narrative device in autobiographical texts associated with superior (or inferior) health outcomes? Does the subjective discovery of particular types of unresolved psychological conflict have relevance for health outcomes? Is the ability to generate psychological resolutions during the writing process associated with well-being? Is coherence in writing associated with superior health outcomes? These are merely a few examples of unexplored directions future studies could pursue utilizing a hybrid approach.

## Endnotes

^1^ I thank an anonymous reviewer for this point.

^2^ While the basic rationale of such quantitative comparisons sounds straightforward, in practice this reasoning depends on a host of methodological and statistical assumptions, some of which are currently under scrutiny in psychology and biomedicine (e.g., Munafó et al. 2017). The details are beyond the scope of this paper, however.
